# Methane and nitrous oxide exchange over a managed hay meadow

**DOI:** 10.5194/bg-11-7219-2014

**Published:** 2014-12-17

**Authors:** L. Hörtnagl, G. Wohlfahrt

**Affiliations:** 1 Institute of Ecology, University of Innsbruck, Austria; 2 European Academy of Bolzano, Bolzano, Italy

## Abstract

The methane (CH_4_) and nitrous oxide (N_2_O) exchange of a temperate mountain grassland near Neustift, Austria, was measured during 2010–2012 over a time period of 22 months using the eddy covariance method. Exchange rates of both compounds at the site were low, with 97% of all half-hourly CH_4_ and N_2_O fluxes ranging between ±200 and ±50 ng m^−2^ s^−1^, respectively. The meadow acted as a sink for both compounds during certain time periods, but was a clear source of CH_4_ and N_2_O on an annual timescale. Therefore, both gases contributed to an increase of the global warming potential (GWP), effectively reducing the sink strength in terms of CO_2_ equivalents of the investigated grassland site. In 2011, our best guess estimate showed a net greenhouse gas (GHG) sink of −32 g CO_2_ equ. m^−2^ yr^−1^ for the meadow, whereby 55% of the CO_2_ sink strength of −71 g CO_2_m^−2^ yr^−1^ was offset by CH_4_ (N_2_O) emissions of 7 (32) g CO_2_ equ. m^−2^ yr^−1^. When all data were pooled, the ancillary parameters explained 27 (42)% of observed CH_4_ (N_2_O) flux variability, and up to 62 (76)% on shorter timescales in-between management dates. In the case of N_2_O fluxes, we found the highest emissions at intermediate soil water contents and at soil temperatures close to 0 or above 14 °C.

In comparison to CO_2_, H_2_O and energy fluxes, the interpretation of CH_4_ and N_2_O exchange was challenging due to footprint heterogeneity regarding their sources and sinks, uncertainties regarding post-processing and quality control. Our results emphasize that CH_4_ and N_2_O fluxes over supposedly well-aerated and moderately fertilized soils cannot be neglected when evaluating the GHG impact of temperate managed grasslands.

## 1 Introduction

Methane (CH_4_) and nitrous oxide (N_2_O) are the most important anthropogenic greenhouse gases (GHGs) after carbon dioxide (CO_2_). Due to their long atmospheric lifetimes of approx. 9 and 131 years (Prather et al., 2012), respectively, both compounds are well mixed in the atmosphere and can influence atmospheric chemistry directly and indirectly. The emission or deposition strength of terrestrial ecosystems is possibly influenced by climate change, which may trigger important feedbacks to the global climate system (Xu-Ri et al., 2012).

CH_4_ has a major influence on the climate and chemistry of the atmosphere (Crutzen and Lelieveld, 2001; Khalil et al., 2007). CH_4_ can react with hydroxyl radicals, resulting in a reduction of the oxidizing capacity of the atmosphere and the production of ozone (O_3_) in the troposphere. Methane can influence the lifetime or production of other atmospheric constituents such as stratospheric water vapor and CO_2_ (Boucher et al., 2009; Collins et al., 2010; Shindell et al., 2009). Its global warming potential over a 100-year lifespan(GWP) and on a per molecule basis is 25 times that of CO_2_ (Forster et al., 2007) or higher when the production of CO_2_ from CH_4_ oxidation is taken into account (Boucher et al., 2009).

The main portion of global CH_4_ originates from single-celled archaea (methanogens) found in anaerobic microsites in the soil, in water-saturated zones rich in carbon and in the digestive systems of ruminants (Baldocchi et al., 2012; Whalen, 2005). CH_4_ is also emitted from organic waste deposits, e.g. manure or from thermogenic and pyrogenic sources (Kirschke et al., 2013). The highest emissions were previously reported from regions with intensive agriculture and animal husbandry (Schulze et al., 2009). Atmospheric CH_4_ increased significantly since the industrial revolution until the end of the 1990s, remained constant for nearly a decade and again began to increase after 2007 (Bousquet et al., 2011; Dlugokencky et al., 2009; Nisbet et al., 2014).

The main sink of methane is through its reaction with the hydroxyl radical OH in the troposphere (Ehhalt and Heidt, 1973). Other minor sinks are methanotrophic bacteria in aerated soils and reactions with atmospheric constituents in the stratosphere and the marine boundary layer (Allan et al., 2007; Cicerone and Oremland, 1988). Previous studies reported reduced CH_4_ deposition in a forest and in a temperate grassland due to elevated CO_2_ (Dubbs and Whalen, 2010; Ineson et al., 1998; Phillips et al., 2001) and increased CH_4_ uptake due to warming in a temperate forest and several subarctic ecosystems (Peterjohn et al., 1994; Sjogersten and Wookey, 2002).

N_2_O can deplete O_3_ in the upper regions and increase O_3_ in the lower regions of the stratosphere (Revell et al., 2012). It can therefore influence tropospheric chemistry by increasing the stratosphere–troposphere exchange of O_3_ and odd nitrogen species, and by increasing OH formation (Prather and Hsu, 2010). Similar to CH_4_, N_2_O has a high warming potential, 298 times that of CO_2_ over a 100-year lifespan (Forster et al., 2007). The dominant source of N_2_O is microbial production through nitrification and denitrification processes in soils, which is fueled by accelerated use of nitrogen fertilizers in agriculture (Davidson, 2009; Fowler et al., 2009). As a consequence of fertilization, agricultural soils are unlikely to act as a sink for N_2_O (Syakila and Kroeze, 2011).

The production of N_2_O by bacteria in soils is controlled by a number of factors, for example, soil water content, temperature and labile carbon availability (Barnard et al., 2005; Holtan-Hartwig et al., 2002; Xu-Ri and Prentice, 2008). Food production was described as the largest single source of N_2_O (Syakila and Kroeze, 2011), while photolysis and oxidation reactions in the stratosphere are the main processes involved in N_2_O depletion (Prather et al., 2012).

Denitrification is an anaerobic process (Zumft and Kroneck, 2007) that is likely exclusively responsible for N_2_O uptake in the soil (Vieten et al., 2008). On a global scale, the uptake of N_2_O by soils may be limited (Chapuis-Lardy et al., 2007). Schlesinger (2013) estimated that the global N_2_O sink in soils is not more than 2% of current estimated sources in the atmosphere. Deposition fluxes to the soil were reported before, e.g., for grasslands, forests, low-nitrogen soils, wetlands and peatlands (Dijkstra et al., 2013; Flechard et al., 2005; Goldberg and Gebauer, 2009a, b; Schlesinger, 2013; Syakila et al., 2010; Wu et al., 2013).

Over managed grasslands, CH_4_ and N_2_O fluxes are characterized by high spatial and temporal variability (Baldocchi et al., 2012; Imer et al., 2013), with emissions of both compounds greatly influenced by land use, management events and animal husbandry. As a consequence, long-term year-round GHG measurements are indispensable when it comes to assessing the effectiveness and feasibility of GHG mitigation strategies.

In this work we present long-term eddy covariance CH_4_ and N_2_O fluxes above a temperate mountain grassland near Neustift, Austria. To this end we investigated 22 months of diurnal, seasonal and interannual exchange rates of both compounds at ecosystem scale and in relation to biotic and abiotic drivers under in situ conditions.

The objective of this study is to (1) quantify eddy covariance CH_4_ and N_2_O fluxes, (2) couple exchange patterns to independent driving variables, (3) determine the annual total GHG balance and (4) compare our findings to previous results from chamber and eddy covariance measurements at ecosystem scale and from laboratory measurements. In-line with these objectives and based on earlier studies, we hypothesized for both compounds that (1) the investigated grassland, due to generally well-aerated soils and modest fertilizer input, is characterized by low fluxes and (2) exchange patterns are predominantly driven by soil parameters. In addition we assumed that (3) despite their low fluxes, CH_4_ and N_2_O exchange significantly contribute to the GHG balance of the meadow.

The study site Neustift, a managed temperate mountain grassland in Austria that is cut three times per year for hay production, was selected because it has been the focus of numerous studies over the last 10 years and is therefore well described in terms of management effects, net ecosystem CO_2_, H_2_O, energy (Brilli et al., 2011; Hammerle et al., 2008; Wohlfahrt et al., 2008b) and volatile organic compound (VOC) exchange (Bamberger et al., 2010, 2011; Brilli et al., 2012; Hörtnagl et al., 2011, 2014; Müller et al., 2010; Ruuskanen et al., 2011).

## 2 Methods

### 2.1 Site description

The study site is an intensively managed meadow in the middle of the flat valley bottom of the Stubai valley in the Austrian Alps, in proximity of the village of Neustift (47° 70′ N, 11° 19′ E) at an elevation of 970 m a.s.l. The climate is humid continental with alpine influences, with an average annual temperature of 6.5 °C; the average annual precipitation amounts to 852 mm. The fetch is homogeneous up to 300 m to the north-northeast (the dominant daytime wind direction) and 900 m to the south-southwest (nighttime) of the instrument tower, parallel to the valley’s orientation. Typically, higher wind speeds and unstable conditions result in a smaller footprint during daytime than during nighttime, where the footprint of the site is larger due to the stable stratification of the atmosphere (Bamberger et al., 2010). The vegetation of the meadow is dominated by a few graminoids (*Dactylis glomerata*, *Festuca pratensis*, *Phleum pratensis*, *Trisetum flavescens*) and forbs (*Ranunculus acris*, *Taraxacum officinale*, *Trifolium repens*, *Trifolium pratense*, *Carum carvi*), while the slopes of the surrounding mountains are covered mainly by coniferous forest. The soil was classified as a Fluvisol (FAO classification) and is approx. 1 m deep, with a thin organic layer (0.001 m), followed by an A horizon that extends down to 0.02 m and a B horizon, best described as a sandy loam. The organic volume fraction of the A horizon is approx. 14%.

Measurements of CH_4_ and N_2_O for this work were conducted from 13 April 2010 to 29 February 2012 (684 days). In each year, the meadow was cut three times, with the first cut on 5 and 6 June, the second cut on 31 July and 1 August and the third cut on 20 and 26 September in 2010 and 2011, respectively. In addition, the meadow was fertilized by manure spreading between 18–22 October in 2010 and on 18–19 October in 2011. The meadow was snow covered from 1 January to 28 February 2010, from 26 November 2010 to 10 March 2011 and from 7 December 2011 to 24 March 2012, resulting in a total of 246 snow days for this analysis. During the measurement campaign, no cows were present on the meadow.

### 2.2 Eddy covariance measurements

The net ecosystem exchange for CH_4_ and N_2_O was calculated by combining the 20 Hz three-dimensional wind speeds quantified by a sonic anemometer (R3IA, Gill Instruments, Lymington, UK) at a height of 2.5 m above ground with the simultaneously detected volume mixing ratios (VMRs) of CH_4_ and N_2_O, which were both measured by a commercially available continuous-wave quantum cascade laser (QCL; CWQC-TILDAS-76-D, Aerodyne, USA). Fluxes were then calculated using the virtual disjunct eddy covariance (vDEC) method proposed by Karl et al. (2002), which is based on the eddy covariance (EC) method (Baldocchi et al., 1988; McMillen, 1988). The intake tube for the QCL was mounted at 0.2 m below the sonic anemometer and displaced laterally perpendicular to the predominating wind direction in order to minimize flux loss due to vertical and longitudinal sensor separation (Massman, 2000). Sample air was drawn from the inlet through a filter (1–2 μm, polytetrafluoroethylene, PTFE) and heated (35 °C) perfluoroalkoxy (PFA) Teflon^®^ tubing (1*/*4″ inner diameter) of 12 m length to the QCL at a flow rate of around 8 SLPM (standard liter per minute; air volume normalized to standard temperature and pressure conditions: 273 K, 1013 hPa). Sonic anemometer data were stored to the hard drive of a personal computer using the Eddymeas software (O. Kolle, Max Planck Institute for Biogeochemistry, Jena, Germany). More details regarding the CO_2_, H_2_O and energy flux measurements are given in Wohlfahrt et al. (2008) and Hammerle et al. (2008).

### 2.3 QCL setup

Ambient air was analyzed for CH_4_, N_2_O and H_2_O at time resolutions of 10 Hz (13 March–16 August 2010), 5 Hz (16–24 August 2010) and 2 Hz (26 August 2010–29 February 2012). The QCL and associated hardware (vacuum pump and Thermo Cube^®^) were housed in a climate-controlled instrument hut next to the field site. During the last 5 min of every half hour, CH_4_-free and N_2_O-free air and air with known, close-to-ambient VMRs were switched into the sampling line to determine zero and span of the QCL, respectively. The QCL was operated at a pressure of 4 kPa using a built-in pressure controller and temperature of the optical bench and housing controlled to 35 °C. The importance of a temperature controlled environment was previously pointed out by Kroon et al. (2007). Fitting of absorption spectra, storing of calculated VMRs, switching of zero-calibration valves, control of pressure lock and other system controls were realized by the TDLWintel software (Aerodyne, USA) run on a personal computer synchronized with the main personal computer collecting anemometer data using the NTP software (Meinberg, Germany).

### 2.4 Despiking

**Figure 1:**
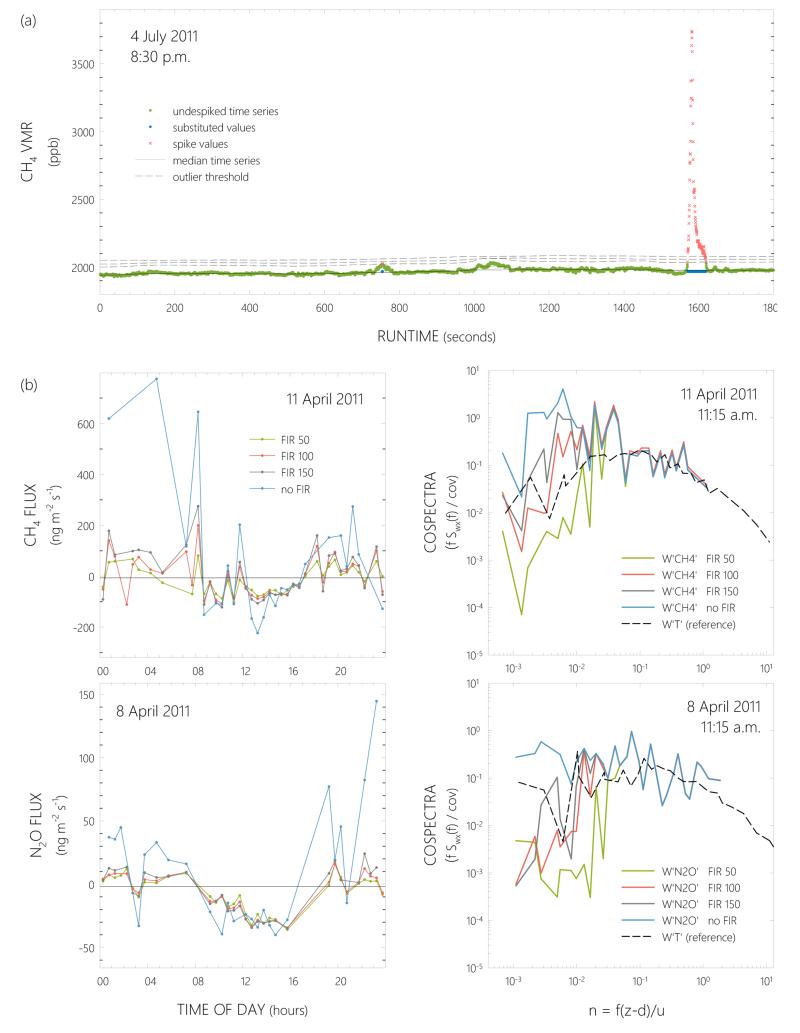
**(a)** Despiking example of 2 Hz methane volume mixing ratios (VMRs) using median filters. **(b)** Diurnal courses (left panels) and normalized co-spectra (right panels) illustrating the effect of high-pass filtering CH_4_ (upper panels) and N_2_O (lower panels) time series with a non-recursive finite impulse response (FIR) filter with different time constants (50, 100 and 150 s). Sensible heat co-spectra are shown in the right panels for reference.

Similar to observations by Baldocchi et al. (2012) for methane, we experienced elevated VMRs of both compounds, but especially CH_4_, at night. We attributed these increased VMRs to atmospheric phenomena in the calm and stable nocturnal boundary layer rather than to elevated biogenic emissions. Therefore, VMRs of both compounds were subjected to a rigorous outlier removal routine before entering flux calculations (Fig. 1a). The despiking method in this study is based on a median filter that runs through each half-hourly VMR time series data point by data point. In comparison to the arithmetic mean, the median value of a time series is relatively insensitive to outlier values. For each half-hourly period, (1) a smoothed time series of the original VMR time series was created. This was done by replacing each original data point with the median value of a moving time window of ±500 values around the respective VMR value. In order to enable the calculation of median values also for data points at the start and end of the measured time series, the first and last 500 values were copied and repeated at the start and end of the smoothed time series, respectively. (2) Each data point in the smoothed time series was then subtracted from the respective measured data point, generating a time series of differences between the two data matrices. (3) When the difference exceeded the empirically determined outlier threshold of 100 ppb, the data point in the measured time series was marked as an outlier. This outlier threshold was tailored to the CH_4_ variability, but also worked well for removing extreme values in the N_2_O time series. (4) The arithmetic mean without these outliers was then calculated and used to (5) replace outliers in the respective half-hourly time series. As turbulent fluctuations for final flux computations are calculated using block averaging, the contribution of these substituted data points to resulting half-hourly fluxes is minor. To better account for natural variability in the time series, three different runs with varying window sizes (±500, 250, 150 values) and outlier thresholds (100, 80, 60 ppb) were performed for each half-hourly period.

During daytime and nighttime, at least one outlier was removed in 30 and 66% of half-hourly CH_4_, but only in 1 and 1% of all recorded N_2_O VMRs, respectively.

### 2.5 Flux calculations

Half-hourly fluxes of CH_4_ (*F*_CH_4__) and N_2_O (*F*_N_2_O_) were then calculated using the virtual disjunct eddy covariance (vDEC) method (Karl et al., 2002) as the covariance between turbulent fluctuations of the vertical wind speed and the VMRs derived from Reynolds averaging of 30 min blocks of data. The time lag between the high-resolution wind data and the disjunct QCL time series was removed using a homemade program, resulting in a subsample of the wind data corresponding to the sampling rate of the QCL. In the same step, CH_4_ and N_2_O fluxes were corrected for the effect of air density fluctuations and laser band broadening following Neftel et al. (2010), using the QCL H_2_O VMR. It was shown previously that flux estimates using the vDEC method are characterized by a larger random uncertainty compared to the true EC, but are unbiased (Hörtnagl et al., 2010). The tubing induced delay time between the wind and the QCL concentration time series was determined in a procedure comprising multiple steps. First, the correlation coefficient between the H_2_O time series measured concurrently by the QCL and a closed-path infrared gas analyzer (Li-7000, LiCor, USA), the data of which were acquired together with the sonic anemometer wind data, was optimized to remove potential time differences between the two personal computers caused by deviating internal clocks, effectively adjusting the starting points of the two time series. Due to generally low values of *F*_CH_4__ and *F*_N_2_O_ at our study site, the determination of lag times between the CH_4_ or N_2_O time series and the wind data was difficult, but worked well between the QCL H_2_O signal and the wind data. Therefore, second, the time delay between the wind components and the QCL H_2_O was determined by identifying the maximum/minimum of the cross-correlation function in a time window of ±7 s. The frequency distribution of this search revealed a peak around 2 s. Third, a second time window of ±2 s (daytime) and ±5 s (nighttime) was then applied around this peak and used for the final lag search between the CH_4_ or N_2_O signal and the vertical wind velocity.

Final fluxes were then calculated using the post-processing software EdiRe (University of Edinburgh). Frequency response corrections were applied to raw fluxes of both compounds, accounting for high-pass (block averaging, finite impulse response filter) and low-pass (lateral sensor separation, dynamic frequency response, scalar and vector path averaging, frequency response mismatch and the attenuation of concentration fluctuations down the sampling tube) filtering according to Massman (2000), using a site-specific co-spectral reference model (Wohlfahrt et al., 2005a). The importance of correcting CH_4_ and N_2_O fluxes for high-frequency losses was shown previously (Kroon et al., 2010c). The high-pass, non-recursive finite impulse response (FIR) filter was applied digitally to account for an overestimation of the flux contributions of low-frequency eddies. The best results were achieved by applying the FIR filter using a Hamming window, whereby time constants of 50 and 100 s for CH_4_ and N_2_O, respectively, sufficiently filtered out unwanted flux contributions at frequencies < 0.05 Hz (Fig. 1b). Missing low-frequencies were then back-corrected based on the site-specific reference model co-spectrum (Wohlfahrt et al., 2005b). Exchange rates of CH_4_ and N_2_O calculated with these settings represent our final best guess fluxes that were used for all analyses in this manuscript.

Two days in April 2011 are used to exemplify the effect of different FIR filters, applied to the CH_4_ and N_2_O time series, on the resulting flux estimates (Fig. 1b). The largest difference between unfiltered and filtered data, as well as between the different filter time constants, was found during nighttime. In contrast, during turbulent conditions, e.g., around noon, fluxes calculated with different time constants exhibited exchange patterns of comparable magnitude (Fig. 1b, left panels). FIR filtering had a larger effect on CH_4_ than on N_2_O fluxes. As an example, over the course of one day unfiltered CH_4_ exchange rates fluctuated between −217 and 780 ng m^−2^ s^−1^ (average: 4 ± 260 ng m^−2^ s^−1^), while best guess fluxes ranged between −96 and 87 ng m^−2^ s^−1^ after FIR filtering (−7 ± 51). Similarly, unfiltered N_2_O fluxes were between −38 and 146 ng m^−2^ s^−1^ (11 ± 46), with best guess fluxes between −33 and 18 ng m^−2^ s^−1^ (−5 ± 15). Co-spectral analyses revealed that lower frequencies of the CH_4_ and N_2_O fluxes were overrepresented compared to the sensible heat flux (Fig. 1b, right panels).

In total, 28 891 raw flux values were calculated for CH_4_ and N_2_O, which corresponds to a data coverage of 88% over the whole measurement period between 13 March 2010 and 29 February 2012. Flux results of each FIR run required separate quality control. When applying a FIR filter with a time constant of 50 / 100 / 150 s to the data, 57 / 55 / 55% of all raw CH_4_ fluxes and 66 / 64 / 63% of all raw N_2_O fluxes passed all quality tests, respectively. However, only 28 and 39% of all raw CH_4_ and N_2_O fluxes, respectively, passed all tests when no FIR filter was used in the flux calculations. Only data that passed all quality tests in a respective scenario were used in the present study. All fluxes in this manuscript are expressed as molecular mass per unit time and ground surface area.

In order to calculate the annual balance of CH_4_ and N_2_O in 2011, the respective quality-controlled half-hourly flux data set was gap-filled. Gaps less than or equal to 2 h were filled by linear interpolation. For the filling of larger gaps a lookup table was generated, using flux data in a time window of 14 days around the missing flux value and *T*_soil_ bin widths of 1 °C. If no lookup table could be generated, e.g. no flux data were available within the time window, the mean diurnal variation (±14 days) was used to fill the gap. For the calculation of the annual GWP of the meadow in Neustift, CH_4_ and N_2_O fluxes were converted to CO_2_ equivalents using the respective compound warming potential as given by Forster et al. (2007).

Instrumentation, data treatment, quality control of CO_2_ and sensible and latent heat fluxes have been described at length by Wohlfahrt et al. (2008) and Hammerle et al. (2008).

### 2.6 Quality control

Half-hourly methane and nitrous oxide fluxes were excluded from the analysis if (i) the deviation of the integral similarity characteristics was larger than 60% (Foken and Wichura, 1996), (ii) the maximum of the footprint function (Hsieh et al., 2000) was outside the boundaries of the meadow, (iii) fluxes were outside a specific range (*F*_CH_4__: ±800 ng m^−2^ s^−1^, *F*_N_2_O_: ±220), (iv) half-hourly VMRs were outside a specific range (CH_4_: 1800–3500 ppb, N_2_O: 280–450 ppb), (v) the stationarity test for the respective flux exceeded 60% (Foken and Wichura, 1996), (vi) the third rotation angle exceeded 10° (McMillen, 1988), (vii) the number of half-hourly VMR values was below 3000 or (viii) more than 20% of data were classified as spikes in any half-hourly period.

### 2.7 Ancillary data

Major environmental parameters were measured continuously at the field site, including air temperature (*T*_air_), soil temperature (*T*_soil_) at 0.05 m depth (TCAV thermocouple, Campbell Scientific, Logan, UT, USA), volumetric soil water content (SWC) (ML2x, Delta-T Devices, Cambridge, UK), soil heat flux (SHF) quantified by means of heat flux plates (three replicates at 0.05 m depth, corrected for the change in heat storage above that depth; HFP01, Hukseflux, Delft, the Netherlands), total photosynthetically active radiation (PAR) (BF3H, Delta-T, Cambridge, UK) and precipitation (52202, R. M. Young, Traverse City, MI, USA). All data were collected continuously by a data logger (CR10X, Campbell Scientific, Logan, UT, USA). The green plant area index (GAI) was assessed (i) in a destructive fashion by harvesting the plant matter of square plots (0.09 m^2^, 3–5 replicates) and subsequent plant area determination (Li-3100, LiCor, Lincoln, NE, USA) and (ii) from measurements of canopy height which were related to destructively measured GAI (Wohlfahrt et al., 2008b). Continuous time series of the GAI were derived by fitting appropriate empirical functions to measured data separately for each growing phase before and after cutting events. A more detailed list of all auxiliary parameters measured at this site is given by Wohlfahrt et al. (2008b) and Hammerle et al. (2008).

### 2.8 Statistical analyses

Statistical analyses were done using Statistica 9 (StatSoft, Inc.), SigmaPlot 12.5 (Systat Software, Inc.) and Excel 2010 (Microsoft, Inc.). The natural logarithm (ln) of the observed daily average CH_4_ and N_2_O fluxes was calculated and used in the simple linear regression (SLR) and multiple linear regression (MLR) analyses as the dependent variable. The partial correlation in the MLR analysis gives the correlation between two variables after controlling for the effect of all other variables in the equation. To determine significant differences between daily average group means in a repeated measures analysis of variance (ANOVA) setting, the unequal n HSD (honest significant difference) post hoc test, a modification of the Tukey’s HSD test, was used. For statistical analyses, only days or half hours where all parameters were available were included. In case of ancillary data, the daily average of the respective parameter was calculated when at least 40 half hours of data were present for the respective day. In comparison, fewer values were available for CH_4_ and N_2_O fluxes and VMRs due to the strict quality criteria. For CH_4_ and N_2_O data, the daily average was regarded as representative for the day when at least 14 half hours were available after quality control. In total 91 and 95% of the presented CH_4_ and N_2_O daily average values, respectively, were calculated from at least 20 half-hourly values. Using daily average values of CH_4_ and N_2_O fluxes in the statistical analyses as opposed to 30 min flux averages reduces random uncertainty (Kroon et al., 2010a).

## 3 Results

**Figure 2:**
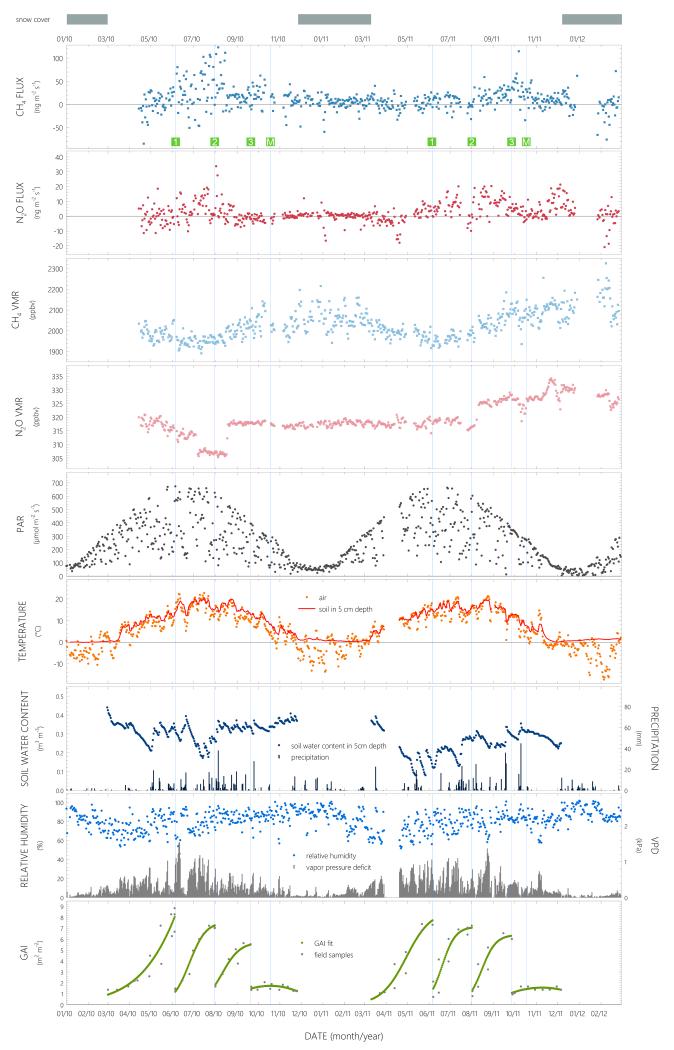
Daily average CH_4_ and N_2_O fluxes and volume mixing ratios (VMRs), photosynthetically active radiation (PAR), air temperature, soil temperature at 5 cm depth, soil water content at 5 cm depth, relative air humidity, vapor pressure deficit, green plant area index (GAI) and daily sums of precipitation over 22 months of measurements between April 2010 and February 2012. Vertical lines show management dates, the numbers 1, 2 and 3 in green squares indicate the first, second and third cutting of the meadow, respectively, while *M* denotes manure spreading.

Daily average values of *F*_CH_4__(*F*_N_2_O_) were calculated for 567 (574) out of 684 days (Fig. 2). While fluxes of both compounds fluctuated around zero towards the end of the vegetation period and during snow cover, net emission and deposition on a daily basis occurred for both compounds during certain time periods. Daily net uptake (negative sign) was recorded on 162 (203) days, whereby time periods characterized by clear deposition were found especially for N_2_O, for example some weeks after snowmelt in spring 2011 (Fig. 2). Highest daily average emissions for CH_4_ (N_2_O) were found around the second cutting of the meadow at the end of July 2010 and amounted to 123.5 (33.4) ng m^−2^ s^−1^. CH_4_ VMRs were the highest during snow cover and the lowest during periods of strong growth (Fig. 2). We attribute the sudden drop of N_2_O concentration values around the first cut in 2010 to a problem with the zero calibration of the QCL. Over the entire 2 years, the median VMR was 2.02 (0.32) ppm for CH_4_ (N_2_O), the median flux amounted to 9.6 (0.9) ng m^−2^ s^−1^ (Fig. 2).

Daily average PAR was found between approx. 40 μmol m^−2^ s^−1^ in winter and 674 μmol m^−2^ s^−1^ in summer, with a median value of 215 μmol m^−2^ s^−1^. In 2010, the yearly average *T*_air_ at the field site of 6.1 °C was colder than the long-term average (2001–2007) of 6.7 °C, while 2011 was warmer than average (7.1 °C). During this study, the maximum daily average *T*_air_ was 22.7 °C in July 2010, the minimum of −17.3 °C was recorded in February 2012 (Fig. 2). *T*_soil_ was similar in both years, approx. 8.5 °C on average and values just above 0 °C when snow covered the ground. SWC was the highest immediately after snow melt, with a maximum daily average value of 0.44 m^3^ m^−3^ at the end of February 2010, and the lowest in May 2011 after a period of only little precipitation (0.08 m^3^ m^−3^). In 2011, SWC was generally low (0.25 m^3^ m^−3^ averaged over the growing season) and significantly lower (*p* < 0.001) than in 2010 (0.32 m^3^ m^−3^). Over the duration of the flux measurements, precipitation was detected on 262 days, amounting to 525 and 537 mm in 2010 and 2011, respectively, and 46 mm over the first 2 months in 2012 (Fig. 2). Relative air humidity (RHA) was around 80% on average over the whole measurement campaign, with minima below 50% in June 2010 (Fig. 2). In 2010 and 2011, the highest vapor pressure deficit (VPD) values of more than 1 kPa were recorded during the warmer months between the end of May and August. GAI was below 1 m^2^ m^−2^ right after snow melt, reached maximum values of up to 8 m^2^ m^−2^ right before the first cut and was then reduced to below 1.5 m^2^ m^−2^ as a consequence of the cutting. GAI maxima before the second and third cut were lower compared to the first cut. Towards the end of the year after the third cut, GAI first increased and later decreased due to vegetation regrowth and senescence, respectively (Fig. 2).

**Figure 3:**
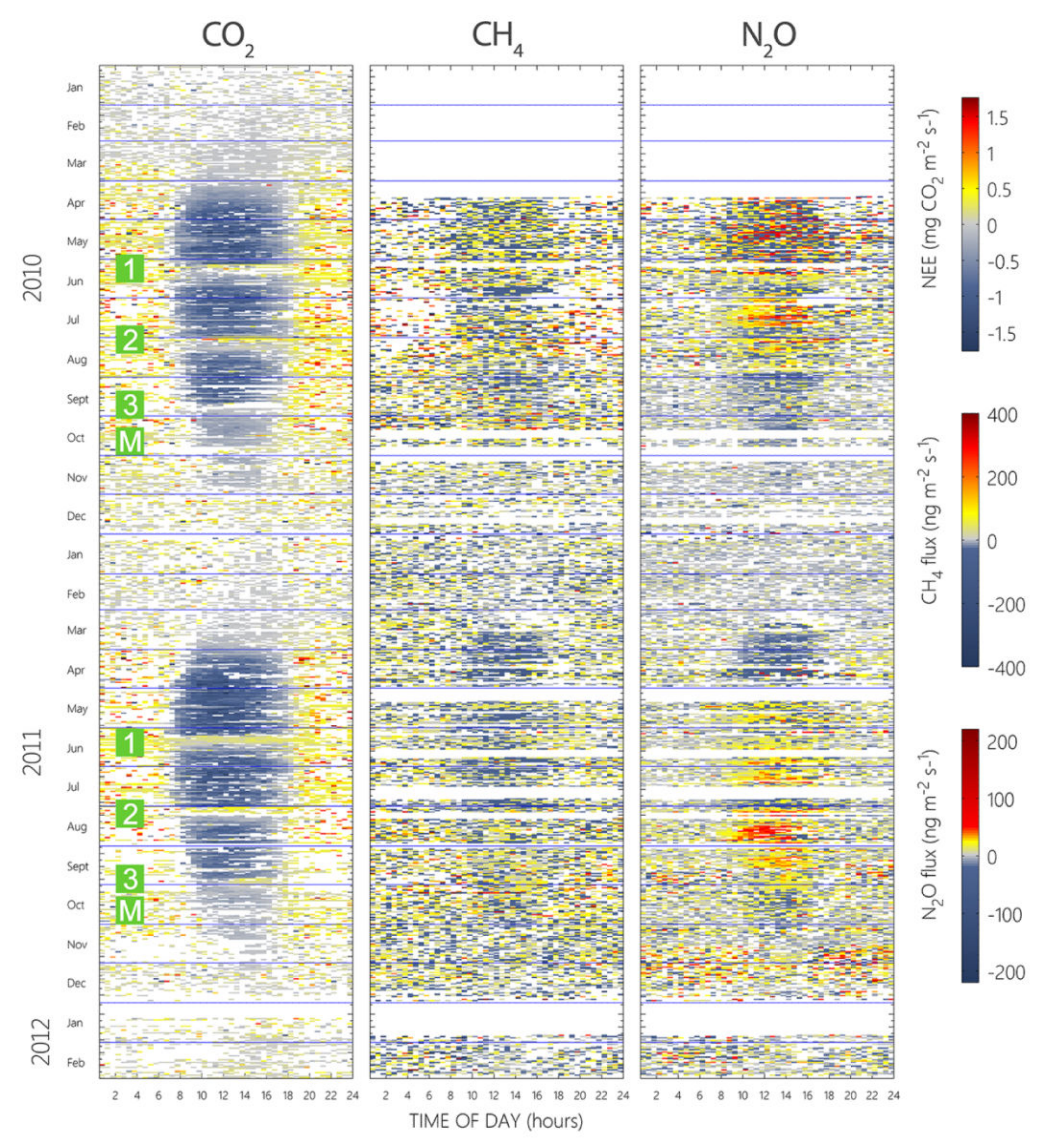
Half-hourly CO_2_, CH_4_ and N_2_O fluxes over 2 years of GHG flux measurements. The numbers 1, 2 and 3 in green squares indicate the first, second and third cutting of the meadow, respectively, while *M* denotes manure spreading. Horizontal blue lines show the start and end of months. White color marks missing data.

The meadow was a source for CO_2_ during snow cover and became a net sink for CO_2_ some weeks after snowmelt and until the first cut (Fig. 3). The cutting event turned the meadow into a CO_2_ source for about 2 weeks before it again became a net sink. This behavior recurred after the second and third cut; however, the CO_2_ uptake after the last cutting was less pronounced than after the previous cuttings. More information about CO_2_ fluxes at the site was given by Wohlfahrt et al. (2008).

Fluxes of both CH_4_ and N_2_O showed high variability on a half-hourly timescale, especially during the first 2 months of the measurements and during the night (Fig. 3). However, 97% of all half-hourly CH_4_ and N_2_O fluxes during the vegetation period were found between ±200 and ±50 ng m^−2^ s^−1^, respectively. During snow-free conditions and including only days not influenced by management events, the average CH_4_ and N_2_O flux was found at 14.0 ± 80.7 and 2.6 ±21.6 ng m^−2^ s^−1^, respectively (Fig. 3). Compared to these undisturbed conditions, average fluxes for both compounds were higher on days where the meadow was influenced by cutting events (17.5 ± 83.7 and 4.8 ± 20.7 ng m^−2^ s^−1^) and lower on days characterized by snow cover (2.1 ± 82.8 and 0.9 ± 20.7). The day of manure spreading, as well as the 2 days thereafter, was covered by our measurements only in October 2011. On the day of fertilization and 2 days later, average N_2_O fluxes were elevated (3.5 ± 17.2 ng m^−2^ s^−1^) when compared to the rest of the same month (1.8 ± 13.6), while CH_4_ fluxes remained virtually unaffected (24.7 ± 91.0 vs. 27.0 ± 88.9). In total, emission fluxes were observed in 56 (57)% of all recorded CH_4_ (N_2_O) half-hour periods (Fig. 3).

**Figure 4:**
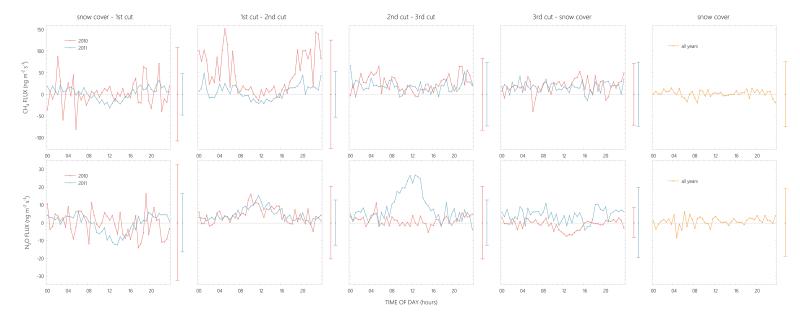
Diurnal cycles of CH_4_ and N_2_O fluxes during different time periods in 2010 and 2011. Whiskers to the right of each plot show the average standard deviation during the respective time period. Management data were excluded from the analysis.

Average diurnal cycles of *F*_CH__4_ and *F*_N_2_O_ were often characterized by high variability with large fluctuations around zero, but followed a clear diurnal cycle during certain time periods (Fig. 4). Methane fluxes showed weak diurnal cycles after snowmelt and before the second cut in 2011, with peak average uptake rates of −31.0 ± 41.4 ng m^−2^ s^−1^ around noon. The uptake of CH_4_ before the first cut coincided with strong N_2_O deposition during daytime, with average peak rates of up to −12.3 ± 23.8 ng m^−2^ s^−1^ in the early afternoon. While CH_4_ fluxes continued to exhibit a very similar deposition pattern up until the second cut, N_2_O fluxes switched in sign and showed a clear diurnal cycle of constant emission during daytime, up to 15.4 ± 18.9 ng m^−2^ s^−1^ on average just before noon. The N_2_O flux pattern after the first and before the second cut was very similar in both years, whereby peak emission rates in 2010 occurred earlier in the day (Fig. 4). In contrast to CH_4_ fluxes, which showed no clear diurnal pattern after the second cut in both years, the meadow constantly emitted N_2_O during daytime and before the third cut in 2011, on average up to 26.8 ± 23.3 ng m^−2^ s^−1^ around noon, while during daytime after the third cut in 2010 N_2_O was transported to the meadow, peak deposition amounted to −7.5 ± 8.8 ng m^−2^ s^−1^ on average. During snow cover, fluxes of both compounds fluctuated around zero (Fig. 4).

When all data were pooled, a MLR analysis explained 27 and 42% of the variability in daily average ln(*F*_CH_4__) and ln(*F*_N_2_O_), respectively, during snow-free conditions (Table 1). Over all years, the partial correlation (PC) of the net ecosystem exchange (NEE) of CO_2_ and *T*_air_ with ln(*F*_CH__4_) was high and positive in sign, while SHF was negatively correlated with ln(CH_4_); all three PCs were highly significant (*p* < 0.001). During shorter time periods in-between, before and after cutting events in single years, the chosen set of parameters explained between 23 and 62% of the observed flux variability, with *r*^2^ being highly significant only once, namely, in a period of high-CH_4_ uptake before the first cut in 2011, with NEE and H as the dominant regressors (Table 1). Explaining the ln(*F*_CH__4_) variance during the same time periods but using data of both years worked best during the vegetation period until the second cut, and again after the third cut until snow cover, explaining up to 40% of observed ln transformed CH_4_ fluxes. The PC of SHF and NEE was significant during the early vegetation period and towards the end of the year, respectively. Latent heat flux (LE) was a significant regressor towards the end of the vegetation period and during snow cover (Table 1). We expanded on these findings by performing a forward stepwise MLR analysis using the same data, effectively reducing the number of variables in the regression equation but yielding similar results. In this analysis NEE, SHF, *T*_air_ and VPD were identified as the most significant regressors (all *p* < 0.05), explaining 25% of the observed ln(*F*_CH__4_) variability over all years excluding snow periods (data not shown). The SLR analysis found highly significant positive correlations for NEE and RHA, and highly significant negative correlations for LE, H and PAR (Table 1).

Generally, the MLR analysis resulted in *r*^2^ being considerably higher for ln(*F*_N_2_O_) than for ln(*F*_CH__4_) (Table 1). The partial correlations were highly significant for multiple regressors. A positive PC was found for the ecosystem fluxes NEE and LE, and in addition for RHA, *T*_air_ and N_2_O VMRs, while significant negative PCs were found for SWC, H and *T*_soil_. All regressors combined were able to explain between 55 and 76% of the ln(*F*_N_2_O_) variance during shorter time periods in single years, with the exception of the time period before the first cut in 2010 when *r*^2^ was found to be statistically not significant (Table 1). The chosen set of parameters performed well with pooled data during the same time periods and especially after the first cut, explaining between 66 and 73% of observed daily average ln(*F*_N_2_O_) values. SWC was the most dominant regressor towards the end of the year, featuring a highly significant, negative PC (Table 1). Similarly, *T*_soil_ was an important parameter in the MLR analysis after the first cut, being first positively and then later negatively correlated with ln transformed N_2_O exchange. Seven parameters were highly significant (*p* < 0.001) in a forward stepwise MLR analysis and explained 41% of the ln(*F*_N_2_O_) variance during snow-free conditions, with *T*_air_, N_2_O VMR, RH, NEE and LE being positively correlated, SWC and H negatively (data not shown). In a simple linear regression 8 out of 11 parameters were significantly correlated with the ln(*F*_N_2_O_), with *T*_air_ and *T*_soil_ as the highest positively and SWC as the highest negatively correlated regressors (Table 1).

**Figure 5:**
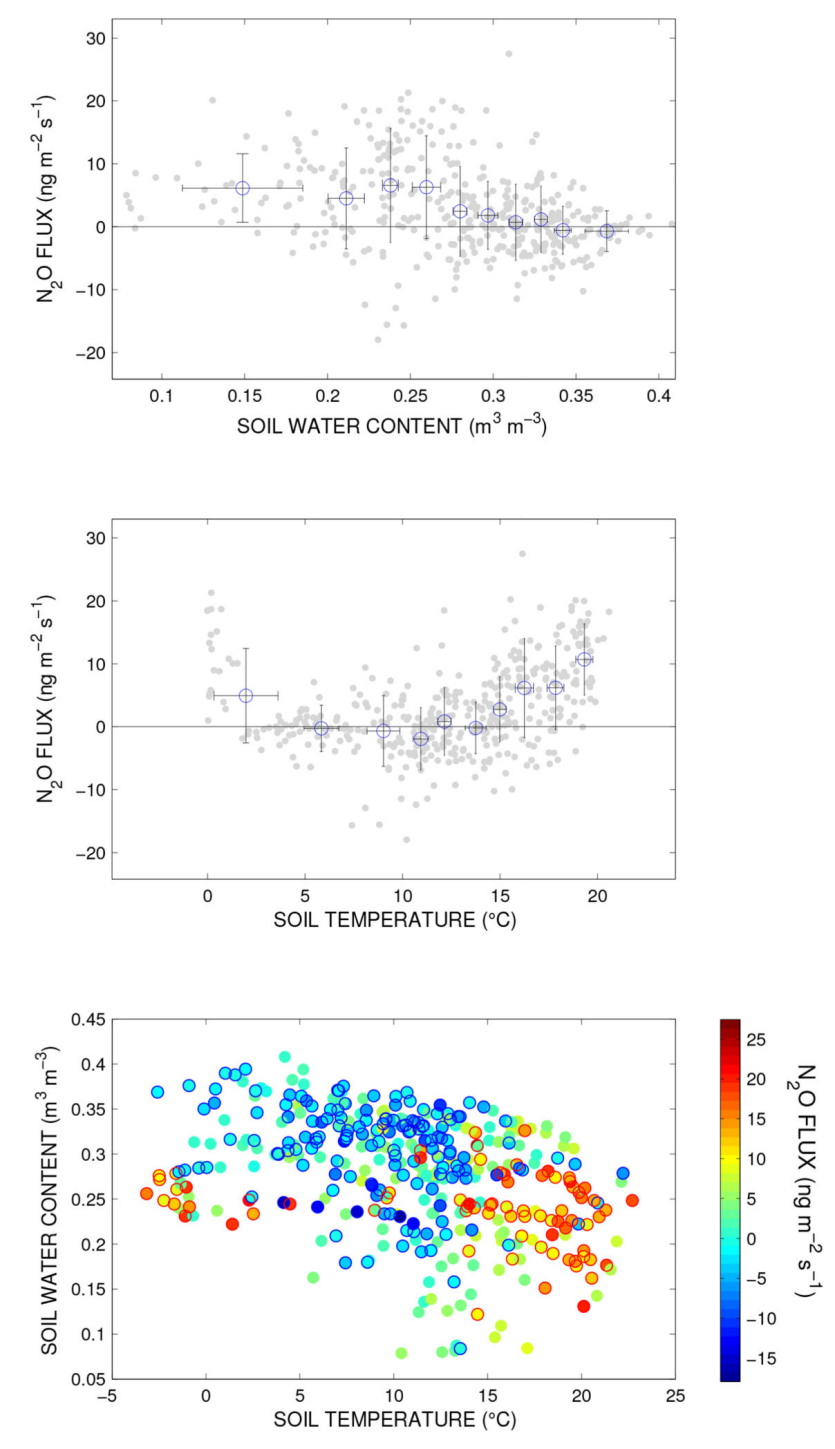
N_2_O daily average fluxes (grey dots) versus soil water content and soil temperature. Blue circles in the upper two panels show bin averages (40 days per bin), with error bars representing the standard deviation within each bin. In the lower panel, fluxes < 0 ng m^−2^ s^−1^ are circled in blue, fluxes > 9 ng m^−2^ s^−1^ are circled in red. Management events were excluded from the analysis.

A closer look at the two most prominent soil related regressors, *T*_soil_ and SWC, and ln(*F*_N_2_O_) under snow-free, undisturbed conditions revealed a clear pattern. Daily average N_2_O exchange showed a bell-shaped relationship with SWC with the highest emissions during periods of intermediate soil water content (Fig. 5, top panel). Even clearer was the correlation between *T*_soil_ and N_2_O flux: days with a daily average *T*_soil_ above 14 °C showed an almost consistent net emission of N_2_O. This was also observed for days where *T*_soil_ was close to 0 °C, whereas N_2_O exchange fluctuated around zero with no clear pattern between 0 and 14 °C (Fig. 5, middle panel). Taking both SWC and *T*_soil_ into account, days characterized by low to intermediate SWC with *T*_soil_ close to 0 °C or above 14 °C generally resulted in a net emission of N_2_O, while deposition was mainly observed during cool conditions with high SWC (Fig. 5, lower panel). In contrast to N_2_O, comparably clear exchange patterns were not found for CH_4_ fluxes.

On a daily average timescale, a repeated measures ANOVA revealed statistically significant differences among environmental conditions on days with net uptake (group *f* −), net emission (*f* +) or close-to-zero exchange (*f* 0) of CH_4_ and N_2_O (Table 2). In case of CH_4_, *T*_air_ was significantly colder on low-flux days than on emission and deposition days. Generally, environmental conditions were most different between high-deposition days and days resulting in emission or close-to-zero exchange of CH_4_ (Table 2). In group *f* −, the ecosystem fluxes LE and H, SHF, PAR, VPD and RHA were all significantly higher compared to *f* + and *f* 0, while also the net uptake of CO_2_ was larger. Although results were less clear for N_2_O fluxes, the meadow tended to act neither as a source or sink on days when air and soil temperatures as well as LE were low (Table 2). In addition, SWC was significantly lower in *f* +, while H was significantly higher on deposition days.

**Figure 6:**
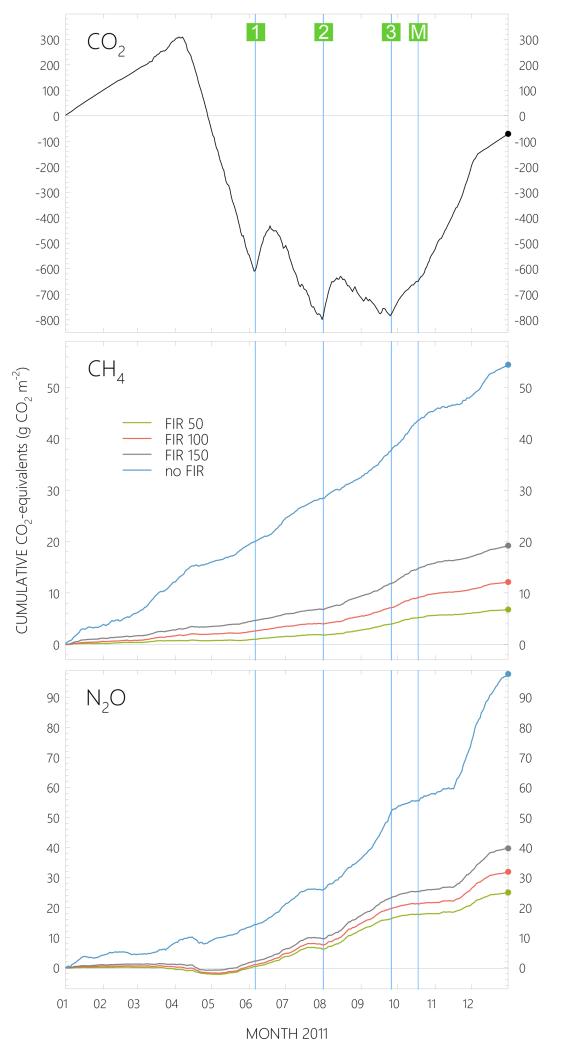
Cumulative GHG fluxes in 2011 expressed as CO_2_ equivalents. The effect of the finite impulse response (FIR) filter with different time constants is shown for CH_4_ and N_2_O budgets. Vertical lines show management dates, the numbers 1, 2 and 3 in green squares indicate the first, second and third cutting of the meadow, respectively, while *M* denotes manure spreading.

Cumulative fluxes for 2011 resulted in a net CO_2_ uptake of −70.5 g CO_2_ m^−2^ (Fig. 6). CH_4_ and N_2_O fluxes were converted to CO_2_ equivalents, with cumulative fluxes being calculated for each of the different FIR filter time constants. In 2011, the meadow acted as a source for both compounds. When no FIR filter was applied, i.e. the overestimation of the low-frequency eddy flux contribution was not corrected for, cumulative methane fluxes amounted to an emission of 54.5 g CO_2_ equ. m^−2^. With FIR filters of varying time constants, cumulative fluxes were considerably lower, in the range of 6.8–19.3 g CO_2_ equ. m^−2^, whereby the lower number was obtained using a FIR filter time constant of 50 s and constitutes our best guess estimate. Results were very similar for N_2_O, the cumulative fluxes of which resulted in a net emission of 97.9 g CO_2_ equ. m^−2^ without FIR filter, and 25.2–39.8 g CO_2_ equ. m^−2^ using filters with different time constants. In case of N_2_O, a time constant of 100 s was considered to give the most representative flux results, yielding 32.0 g CO_2_ equ. m^−2^ over the whole year (Fig. 6).

The total GHG budget can be calculated by summing up the different cumulative contributions of CO_2_, CH_4_ and N_2_O. Based on the best guess estimates, the meadow acted as a GHG sink (−31.7 g CO_2_ equ. m^−2^) in 2011. However, when no FIR filter was applied to neither CH_4_ nor N_2_O data, the sum of the two compound fluxes more than compensated for the sink effect of CO_2_, turning the meadow into a GHG source (81.9 g CO_2_ equ. m^−2^; Fig. 6).

## 4 Discussion

### 4.1 Methane

It was shown recently that plants do not contain a known biochemical pathway to synthesize methane (Nisbet et al., 2009), a finding that contradicts observations of methane emissions from terrestrial plants under aerobic conditions in an earlier study (Keppler et al., 2006). Methane emissions from plant tissue may be due to the transpiration of water that contains dissolved CH_4_ or due to the abiotic breakdown of plant material as a consequence of high-UV stress conditions (Nisbet et al., 2009), but the contribution of terrestrial plants to the global methane emission is considered to be small (Dueck et al., 2007). Based on these earlier findings it is feasible to regard observed eddy covariance emission fluxes in this study as a direct (methanogen microorganisms) or indirect (transpiration of soil CH_4_) consequence of processes in the soil, an important player in the global methane cycle (Kirschke et al., 2013; Smith et al., 2000).

Therefore, one might expect clear relationships between soil environmental parameters such as temperature or moisture and CH_4_ exchange, which were also reported by other studies (Dijkstra et al., 2013; Hartmann et al., 2010; Imer et al., 2013; Jackowicz-Korczyński et al., 2010; Kroon et al., 2010b; Liebig et al., 2009; Rinne et al., 2007; Schrier-Uijl et al., 2010). However, when all data were pooled no clear correlation between soil parameters and eddy covariance CH_4_ exchange at the grassland site in Neustift was observed. Although the explanatory power of *T*_soil_ in the MLR was relatively high and significant between the first and second cutting of the meadow in 2011 – a period when small quantities of CH_4_ were taken up by the meadow around noon – no consistent relationship between soil parameters and the CH_4_ flux was observed (Table 1). SHF was significantly higher on days with net deposition compared to zero-flux and net emission days (Table 2), which might be an indication of soil processes as possible drivers for observed exchange patterns. The partial correlations of SWC with CH_4_ exchange, however, were statistically not significant throughout the measurement campaign and close to 0 °C when all data were pooled (Table 1). This is in contrast to chamber studies that identified soil moisture as a key driver for methane exchange (e.g. Dijkstra et al., 2013).

One explanation for this lack of correlation between soil parameters and methane fluxes might be that half-hourly eddy covariance fluxes represent an integral signal, averaged over 30 min over a possibly heterogeneous area of methane sources and covering both hot spots of high-methane emissions and areas of relatively high uptake within the same flux footprint (Baldocchi et al., 2012). Therefore, SWC may be high in certain patches of the meadow and create environmental conditions conducive for methanogenic microorganisms, but low in other microsites across the grassland. Half-hourly fluxes reflect this heterogeneity across the footprint to a varying degree, mainly depending on wind direction, wind speed and atmospheric stability. In addition, the direct effect of certain drivers on CH_4_ exchange may smear out at ecosystem scale, especially if associated fluxes are generally low. Recently, Yvon-Durocher et al. (2014) found an average temperature dependence of CH_4_ emissions from aquatic, wetland and rice paddy ecosystems similar to that of CH_4_ production derived from pure cultures of methanogens and anaerobic microbial communities in the laboratory. No such relationship was found in the present study, which may be a direct consequence of a heterogeneous footprint with regards to CH_4_ sources and generally low CH_4_ fluxes at the measurement site in Neustift.

The observation of weak CH_4_ uptake around noon between March and July 2011 (Fig. 2) is most likely a consequence of methanotrophic microorganisms in the soil, a process enhanced by increased soil temperature. However, it is difficult to observe this temperature dependence at ecosystem scale, as the whole footprint regardless of emission/deposition hot spots is sampled. In addition, it was shown that both methanotrophic and methanogenic activity in the soil are temperature dependent (von Fischer and Hedin, 2007; Yavitt et al., 1995), whereby the latter tends to be more responsive to temperature (Topp and Pattey, 1997). Imer et al. (2013) reported nearly consistent methane uptake throughout the year except for winter at three different grassland sites along an altitudinal and management gradient using static chambers, with flux rates of generally below 10 ng m^−2^ s^−1^. Three pastures investigated by Liebig et al. (2009) were identified as minor CH_4_ sinks.

Daily average CH_4_ emissions in this study generally ranged up to 100 ng m^−2^ s^−1^ and were relatively similar to eddy covariance results over a drained and grazed peatland pasture during dry periods, when fluxes were often below 160 ng m^−2^ s^−1^ (Fig. 2; Baldocchi et al., 2012). However, the maximum CH_4_ flux and concentration of more than 5700 ng m^−2^ s^−1^ and 3500 ppb, respectively, at the peatland site were much higher than the 128 ng m^−2^ s^−1^ and 2300 ppb recorded at Neustift. Higher maximum methane fluxes were also observed by Schrier-Uijl et al. (2010) over a grass ecosystem on peat (1604 ng m^−2^ s^−1^).

In comparison to CO_2_ and energy fluxes, there are only a few long-term EC methane exchange studies. However, year-round measurements are indispensable for accurately estimating the CH_4_ budget of an ecosystem. Baldocchi et al. (2012) reported a 3-year-mean annual methane efflux at a peatland pasture of 11.6 ± 9.0 g m^−2^ yr^−1^ without any discrimination for cattle or elongated footprints during the night, and 3.6 ± 1.9 g m^−2^ yr^−1^ when only daytime data representing the well-drained portion of the pasture, additionally filtered for favorable wind directions and the presence of cows, were used. This latter number is relatively similar to the methane efflux of 2.1 g m^−2^ yr^−1^ in Neustift in 2011. In comparison, Hendriks et al. (2007) reported 14.2 ± 26.1 g m^−2^ yr^−1^ from the relatively dry portions of an abandoned peat meadow using chamber measurements, and 42.5 ± 27.7 g m^−2^ yr^−1^ when the whole meadow, including water-saturated land and ditches, was considered. Mander et al. (2010) conducted a literature survey and reported median fluxes of 0.16 g m^−2^ yr^−1^ for fertilized grasslands on hydromorphic soils in Estonia, similar to Neustift (0.27 g m^−2^ yr^−1^). Methane emissions reported by Merbold et al. (2014) from a grassland after restoration where 1 order of magnitude higher (3.6 g m^−2^ yr^−1^). Using eddy covariance measurements, methane emissions between 24 and 29 g m^−2^ yr^−1^ were reported from a subarctic peatland (Jackowicz-Korczyński et al., 2010), 12.6 g m^−2^ yr^−1^ from a boreal fen (Rinne et al., 2007) and 16.5 g m^−2^ yr^−1^ from a managed fen meadow (Kroon et al., 2010b).

Baldocchi et al. (2012) reported mean diurnal patterns characterized by the lowest methane efflux densities during midday and elevated methane emissions throughout the night, a pattern very similar to Neustift during certain time periods, e.g. between the first and second cut in 2010 (Fig. 4). We mainly attributed this observation to meteorological factors, i.e. intermittent exchange during calm and stable nighttime conditions, which was also the reasoning behind the outlier handling in our despiking procedure (Fig. 1a). Another reason might be the preferential sampling of an elevated methane source in combination with a larger nighttime footprint as described by Baldocchi et al. (2012). It is possible that methane emissions from a small stream and adjacent wet patches of the meadow, that are normally not part of the footprint, have contributed disproportionally to observed methane emissions. Unfortunately, we lack detailed high-resolution spatial data (e.g. vegetation, soil) about small areas and patches within the sampled flux footprint in Neustift, which would be required for a meaningful footprint analysis. Therefore, we are currently not able to further discuss potential emission hotspots, their impact on calculated CH_4_ balances and the problem of possibly preferential sampling within this manuscript. Hot spot footprint analysis merits its own research and would provide important insights in how to interpret eddy covariance flux data.

Several studies reported that 81–90% of the total annual methane emissions occurred during the snow-free period or between spring and autumn (Jackowicz-Korczyński et al., 2010; Rinne et al., 2007), which is very similar to Neustift in 2011, where 84% of the yearly net CH_4_ emissions occurred during snow-free conditions.

### 4.2 Nitrous oxide

Despite occasional uptake, the meadow was a source of N_2_O, in accordance with previous studies over managed grasslands. Half-hourly emission rates of N_2_O, mostly below 50 ng N_2_O m^−2^ s^−1^, were similar to exchange rates reported by Neftel et al. (2010) for an experimental farm site and Imer et al. (2013) from a mountain rangeland. N_2_O fluxes in 2011 amounted to an emission of 107 mg m^−2^ yr^−1^. For comparison, Mander et al. (2010) reported approx. 94 and 723 mg m^−2^ yr^−1^ for unfertilized and fertilized grasslands, respectively. Considerably higher emissions were found by Kroon et al. (2010b) for a managed fen meadow (2.4 g N_2_O m^−2^ yr^−1^), and by Merbold et al. (2014) for a grassland after restoration (4.6 g m^−2^ yr^−1^).

Many of the observations made for CH_4_ were also valid for N_2_O, with generally low fluxes, a possibly heterogeneous flux footprint with respect to emission/deposition hot spots and soil processes as the driving force behind N_2_O exchange patterns. In contrast to CH_4_ exchange, N_2_O fluxes on a daily scale could be well explained by environmental parameters during specific time periods. The important role of temperature in soil processes was shown previously, as N mineralization, nitrification, denitrification and N_2_O emissions all increase with temperature (Barnard et al., 2005), while reduced soil moisture as a result of high air temperatures and increased plant transpiration can decrease N_2_O emissions (Li et al., 1992). These findings are comparable to observations in the present study, where N_2_O exchange tended to emission during warm and relatively dry soil conditions (Fig. 5, lower panel).

N_2_O consumption in the soil occurs when N_2_O reduction exceeds N_2_O production (Chapuis-Lardy et al., 2007). Soil water is probably the key driver regulating N_2_O consumption in soils, as it can act as a temporary storage body that entraps N_2_O, effectively hindering its diffusion from the soil matrix to the surface. As a consequence, the time for potential reduction of N_2_O to N_2_ through anaerobic denitrification is increased (Clough et al., 2005). This can result in a low N_2_O */* N_2_ ratio during wet conditions, which favors N_2_O consumption (Ruser et al., 2006; Wu et al., 2013). These observations agree with our findings at ecosystem scale. When all data were pooled, N_2_O uptake was the highest during relatively wet conditions (Fig. 5, top panel) and SWC was significantly lower on days with clear net emission of N_2_O (Table 2). The latter finding is further highlighted by a clear positive correlation between daily average ln(*F*_N_2_O_) and *T*_soil_ in the soil temperature range 12–16 °C as long as SWC was low (data not shown).

In October 2011, manure application resulted in a pulse of N_2_O emissions 1 day later, after which fluxes rapidly decreased and reached pre-fertilization rates 2 days after manure spreading. Similar behavior of N_2_O fluxes returning to background levels within 2–6 days after fertilization has been observed by Jones et al. (2011) for a Scottish grassland and Neftel et al. (2010) for an experimental farm site. Pulses of N_2_O emissions after fertilizer application were also described in other studies (e.g. Granli and Bockman, 1994; Jones et al., 2011) and might be the result of animal manure – the most concentrated form of anthropogenic N input (Davidson, 2009) – directly fueling nitrifying and denitrifying bacteria in the soil, which are most active when N is abundant (Firestone and Davidson, 1989). Over the weeks following fertilization, N_2_O emissions increased with air temperature, which is in-line with the temperature dependence of the involved processes. We observed a sharp increase of N_2_O emissions once the daily average air temperature fell below the freezing point, approx. 4 weeks after manure spreading in November 2011. During this time period the meadow remained snow free, with soil temperatures close to 0 °C. The combination of reduced plant metabolism (low nitrate demand by plants) and prior manure spreading could result in an abundance of soil nitrate at the end of the vegetation period. Wertz et al. (2013) showed that denitrification can still occur at very low temperatures and even below the freezing point when nitrate and C are present. The observation of high-N_2_O emissions from frozen or nearly frozen soil was also made by earlier studies (Röver et al., 1998; Teepe et al., 2001).

Production and subsequent emission of N_2_O remained high after the beginning of the snow cover in December 2011. Zhu et al. (2005) described a similar situation where microbial activity in the soil of a lowland tundra did not cease during snow cover and N_2_O continuously diffused to the atmosphere through the snowpack. In Neustift, high N_2_O emissions were not observed 1 year earlier during similar conditions.

### 4.3 Global warming potential

The availability of year-round data allows for the calculation of a yearly GWP balance over a specific ecosystem. In this study, year-round CH_4_, N_2_O and CO_2_ flux data were available for 2011. When expressing the net exchange of the three compounds in terms of CO_2_ equivalents and adding up these different contributions, the resulting GWP of the meadow in Neustift was −32 g CO_2_ equ. m^−2^ yr^−1^ in 2011, whereby a yearly NEE of −71 g CO_2_ m^−2^ yr^−1^ was offset by CH_4_ and N_2_O emission of 7 and 32 g CO_2_ equ. m^−2^ yr^−1^, an offset of approx. 55%.

Liebig et al. (2009) investigated 3 years of CH_4_ and N_2_O static chamber fluxes, soil organic carbon change, CO_2_ emissions associated with N fertilizer production and CH_4_ emissions from enteric fermentation for three grazing management systems. The resulting net GWP between −78 and 40 g CO_2_ equ. m^−2^ yr^−1^ is similar to results in this study. Hendriks et al. (2007) reported −86 g CO_2_ equ. m^−2^ yr^−1^ from an abandoned peat meadow. Merbold et al. (2014) gave the full GHG flux budget of an intensively managed grassland after restoration, including ploughing. GHG emissions reported in their study were much higher than in Neustift, amounting to 2.9 kg CO_2_ equ. m^−2^, and relatively similar to the balance of 1.6 kg CO_2_ equ. m^−2^ found by Kroon et al. (2010b) for a managed fen meadow. Zona et al. (2013) reported a GHG balance of −260 g CO_2_ equ. m^−2^ yr^−1^ for a poplar plantation in 2011, taking into account CO_2_ fluxes of −351 g CO_2_ equ. m^−2^ yr^−1^, and CH_4_ and N_2_O fluxes of 49 and 42 g CO_2_ equ. m^−2^ yr^−1^, respectively, with CH_4_ and N_2_O offsetting the NEE sink by 26%. Soussana et al. (2007) investigated the GHG budget of nine European grassland sites over 2 years, covering a major climatic gradient and a wide range of management regimes. On average, the investigated grassland plots were a net sink of −879 g CO_2_ m^−2^ yr^−1^, and a net source of 117 and 51 g CO_2_ equ. m^−2^ yr^−1^ for CH_4_ and N_2_O, respectively, with emissions of the latter two compounds resulting in a 19% offset of the NEE sink activity. Tian et al. (2014) reported offset ratios of 73% for the whole North American continent, with the grassland GWP being nearly neutral.

Rinne et al. (2007) reported a GWP balance of +108 g CO_2_ equ. m^−2^ when taking into account CO_2_ and CH_4_ fluxes from a boreal fen, with respective fluxes amounting to −156 and +264 g CO_2_ equ. m^−2^. Although the GWP calculated from CO_2_ and CH_4_ fluxes was much lower in Neustift (−64 g CO_2_ equ. m^−2^), the situation was similar in that the carbon uptake of the meadow through CO_2_ was partially offset by carbon loss through CH_4_ emissions. The number for Neustift may change drastically on a year-to-year basis, as the meadow can act both as a source and sink of CO_2_ (Wohlfahrt et al., 2008), while it is supposedly a constant source of CH_4_. Dijkstra et al. (2013) used static chambers to calculate the GWP for 5 years of CO_2_ and CH_4_ data in a semiarid grassland, ranging between −3 and −6 g CO_2_ equ. m^−2^.

## 5 Conclusions

The grassland site in Neustift is characterized by low fluxes of CH_4_ and N_2_O. Although the meadow can act as a source and sink for both compounds during certain time periods, it is a clear source of CH_4_ and N_2_O on an annual timescale. As a consequence, both gases contribute to an increase of the GWP, effectively reducing the sink strength in terms of CO_2_ equivalents.

Our analyses showed that daily average N_2_O exchange during most of the vegetation period can be well explained with simultaneously recorded ancillary data, especially in the time period after the first cut in June up until snow cover towards the end of the year. In contrast, modeling daily average exchange with the same ancillary data worked considerably worse for CH_4_, a finding that suggests the possibility of a more heterogeneous footprint in regard to methane sources and sinks. For both compounds it was not possible to single out one driving variable as the most important one, which is to be expected due to the nature of the eddy covariance flux signal in combination with generally low CH_4_ and N_2_O fluxes at the investigated grassland site.

In comparison to CO_2_, H_2_O and energy fluxes, the interpretation of CH_4_ and N_2_O exchange is challenging due to uncertainties regarding post-processing, quality control and footprint heterogeneity. Knowledge about emission and deposition hotspots within the footprint area would allow for a more comprehensive interpretation of the bulk EC flux. Additional information about GHG producing and consuming patches within the flux footprint could be achieved, for example, via chamber measurements; another possibility would be to perform a detailed statistical analysis of EC fluxes and underlying footprint information in combination with detailed spatial data of the sampled area.

We conclude that CH_4_ and N_2_O fluxes over supposedly well-aerated and moderately fertilized soils cannot be neglected when evaluating the GHG impact of temperate managed grasslands. Both compounds can significantly influence the GWP balance of a meadow and be determining if a grassland is acting as a source or sink of CO_2_ equivalents. In order to reliably assess GHG budgets on a local and global scale, long-term measurements of CH_4_ and N_2_O fluxes in combination with CO_2_ exchange are necessary, especially over ecosystems that are normally characterized by low GHG fluxes. In addition, we recommend to carefully check flux results and underlying co-spectra for an overestimation in the low-spectral range and correct for this effect if necessary.

## Figures and Tables

**Table 1:** Partial correlations of a multiple linear regression analysis and correlation coefficients (*r*) of a simple linear regression analysis using daily average values of ln transformed CH4 (*F*_CH_4__) and N_2_O (*F*_N_2_O_) flux rates as dependent variables and air temperature (T_air_), soil temperature (T_soil_) and soil water content (SWC) at 5 cm depth, soil heat flux (SHF), net ecosystem CO_2_ exchange (NEE), latent heat flux (LE) and sensible heat flux (H), photosynthetically active radiation (PAR), vapor pressure deficit (VPD), relative air humidity (RHA) and CH_4_ (N_2_O) volume mixing ratios (VMRs) as independent variables. Management events were excluded from the analysis. Bold numbers highlight *p* <0.05, except bold underlined numbers resulted in *p* <0.001. Results shown for the vegetation period do not include time periods with snow cover on the meadow.

Multiple linear regression partial correlations															Simple linear regression *r*

	vegetation period	snow melt–first cut			first cut–second cut			second cut–third cut			third cut–snow cover			snow cover	vegetation period
	2010–11	2010	2011	2010–2011	2010	2011	2010–2011	2010	2011	2010–2011	2010	2011	2010–2011	2010–2012	2010–2011
ln(*F*_CH_4__)															

*T*_air_	**0.19**	0.07	0.07	**0.25**	−0.02	−0.35	0.11	−0.05	0.32	0.02	0.20	0.01	−0.02	0.17	**0.13**
*T*_soil_	−0.04	0.10	−0.07	−0.16	−0.13	**0.57**	0.10	−0.08	−0.12	−0.09	0.01	0.15	0.11	−0.11	**0.16**
SWC	0.07	0.06	−0.24	0.04	−0.20	−0.13	0.13	0.03	0.33	0.05	0.33	−0.05	−0.09	−0.13	**0.10**
SHF	**−0.22**	−0.14	−0.14	**−0.26**	0.12	0.22	−0.16	0.02	−0.28	0.01	−0.29	−0.08	−0.10	0.04	−0.09
NEE	**0.20**	0.12	**0.38**	0.18	0.24	0.10	0.19	−0.04	0.05	0.01	0.33	0.18	**0.32**	0.20	**0.30**
LE	−0.05	−0.16	−0.17	−0.12	0.09	−0.23	−0.05	−0.21	0.10	−0.17	0.20	**0.48**	**0.44**	**0.28**	**−0.19**
H	−0.06	−0.08	**−0.38**	−0.13	−0.25	−0.03	0.09	−0.08	0.13	0.10	0.01	−0.33	−0.18	−0.09	**−0.19**
PAR	0.10	0.23	0.16	**0.25**	−0.08	−0.16	0.00	0.25	−0.20	0.06	−0.17	−0.07	−0.13	0.00	**−0.19**
VPD	−0.07	0.08	0.02	−0.09	−0.01	0.10	−0.01	0.20	−0.26	0.19	−0.11	−0.16	−0.08	−0.12	−0.09
RHA	0.03	0.12	0.07	0.06	0.12	0.05	0.05	0.30	−0.28	0.21	−0.31	0.03	0.02	−0.08	0.23
CH_4_ VMR	0.01	0.08	0.00	0.02	0.15	**0.39**	0.06	**−0.35**	0.11	−0.15	0.35	−0.12	−0.11	0.01	0.02
multiple *r*^2^	**0.27**	0.31	**0.54**	**0.20**	**0.43**	**0.62**	**0.36**	0.41	0.23	0.18	**0.55**	**0.53**	**0.40**	0.22	
N	356	47	67	114	50	36	86	44	40	84	35	37	72	82	365–397

ln(*F*_*N*_2_O_)															

*T*_air_	**0.14**	−0.04	**0.27**	0.03	0.28	−0.06	0.03	0.10	0.14	0.21	0.03	0.05	0.05	0.17	**0.29**
*T*_soil_	**−0.11**	0.09	−0.16	0.06	−0.16	0.22	**0.30**	−0.07	−0.06	**−0.27**	−0.22	−0.18	**−0.33**	−0.12	**0.24**
SWC	**−0.24**	−0.13	−0.15	**−0.24**	−0.18	−0.27	−0.21	**−0.38**	−0.31	**−0.51**	0.01	**−0.45**	**−0.47**	−0.05	**−0.33**
SHF	−0.02	0.03	−0.23	0.02	−0.26	0.16	−0.11	−0.12	−0.11	−0.14	**0.42**	0.19	0.24	−0.10	**0.15**
NEE	**0.23**	−0.16	**0.31**	0.13	0.10	−0.10	0.10	**0.35**	0.24	**0.32**	0.32	−0.12	0.09	0.02	0.00
LE	**0.19**	−0.16	−0.11	−0.08	−0.10	0.07	−0.03	0.04	0.19	0.11	**0.41**	0.05	0.17	0.18	**0.22**
H	**−0.14**	−0.24	−0.16	**−0.23**	−0.08	−0.20	−0.14	**0.45**	0.00	0.18	0.22	−0.10	0.13	**−0.25**	**−0.20**
PAR	−0.02	0.21	0.21	0.13	**0.37**	−0.03	0.22	−0.22	0.18	0.02	−0.37	−0.04	**−0.32**	0.06	0.05
VPD	0.01	−0.24	−0.11	−0.11	0.09	−0.04	0.13	0.26	0.05	**0.23**	**−0.47**	−0.03	−0.17	−0.10	**0.16**
RHA	**0.24**	−0.21	0.18	0.01	**0.45**	0.03	**0.33**	**0.37**	0.23	**0.37**	**−0.60**	0.07	−0.13	0.00	0.08
N_2_O VMR	**0.25**	−0.05	−0.11	0.02	**0.39**	0.28	**0.26**	−0.15	0.11	−0.13	−0.26	−0.06	−0.04	**0.39**	**0.17**
multiple *r*^2^	**0.42**	0.19	**0.55**	**0.26**	**0.76**	**0.73**	**0.66**	**0.72**	**0.56**	**0.68**	**0.73**	**0.68**	**0.73**	**0.44**	
N	360	49	67	116	50	36	86	44	41	85	36	37	73	83	369–401

**Table 2:** Daily average means in three different groups of daily net CH_4_ (N_2_O) exchange. Significant differences between group means were determined in a repeated measures ANOVA setting, using the unequal n HSD post hoc test. Group labels to the right of a given group mean show to which flux group the respective value was significantly different. Bold numbers mark group means that were significantly different from one other group, while bold underlined numbers denote group means that were significantly different from both other groups. *f*+ daily average CH_4_ (N_2_O) emission fluxes >3 (0.4) ng m^−2^ s^−1^, *f*0 fluxes between 3 (0.4) and −3 (−0.4) ng m^−2^ s^−1^, *f*− deposition fluxes < −3 (−0.4) ng m^−2^ s^−1^.

Mean values, standard deviations and significant differences													

Compound flux class	Unit	CH_4_						N_2_O					

		*f*+		*f* −		*f*0		*f*+		*f* −		*f*0	
*T*_air_	°C	**9.2 ± 7.0**	***f*0**	**9.6 ± 6.0**	***f*0**	**5.9 ± 8.5**	***f*+**, ***f*−**	**10.1 ± 7.5**	***f*0**	**8.4 ± 5.1**	***f*0**	**4.2 ± 7.3**	***f*+**, ***f*−**
*T*_soil_	°C	10.8 ± 6.5		10.9 ± 5.3		8.2 ± 6.7		**11.5 ± 6.8**	***f*0**	**10.1 ± 4.8**	***f*0**	**6.5 ± 5.5**	***f*+**, ***f*−**
SWC	m^3^ m^−3^	0.29 ± 0.06		0.28 ± 0.06		0.29 ± 0.09		**0.27 ± 0.07**	***f*−**, ***f*0**	**0.31 ± 0.05**	***f*+**	**0.32 ± 0.06**	***f*+**
SHF	W m^−2^	**1.0 ± 6.9**	***f* − **	**3.9 ± 6.6**	***f*+**, ***f*0**	**0.5 ± 6.4**	***f* − **	**2.2 ± 7.3**	***f*0**	1.4 ± 6.0		**−1.5 ± 6.1**	***f*+**
NEE	μg CO_2_ m^−2^ s^−1^	**−70 ± 224**	***f* − **	**−220 ± 229**	***f*+**	−119 ± 220		−106 ± 246		−141 ± 220		−40 ± 172	
LE	W m^−2^	**55 ± 53**	***f* − **	**86 ± 58**	**f+**, ***f*0**	**50 ± 57**	***f* − **	**67 ± 64**	***f*0**	**64 ± 44**	***f*0**	**30 ± 37**	**f+**, **f−**
H	W m^−2^	**6.9 ± 22.0**	***f* − **	**20.2 ± 22.7**	***f*+**, ***f*0**	**8.8 ± 19.8**	***f* − **	**7.5 ± 22.8**	***f* − **	**16.7 ± 21.9**	***f*+**, ***f*0**	**4.4 ± 16.8**	***f* − **
PAR	μmol m^−2^ s^−1^	**271 ± 158**	***f* − **	**372 ± 169**	***f*+**, ***f*0**	**250 ± 168**	***f* − **	293 ± 180		**314 ± 149**	***f*0**	**217 ± 139**	***f* − **
VPD	kPa	**0.33 ± 0.28**	***f* − **	**0.42 ± 0.26**	***f*+**, ***f*0**	**0.28 ± 0.29**	***f* − **	0.36 ± 0.30		0.35 ± 0.25		0.23 ± 0.22	
RHA	%	**81 ± 10**	***f* − **	**75 ± 10**	***f*+**, ***f*0**	**82 ± 11**	***f* − **	**81 ± 10**	***f* − **	**77 ± 11**	***f*+**	82 ± 12	
VMR	ppb	2014 ± 59		2004 ± 53		2021 ± 60		**319 ± 6**	***f* − **	**317 ± 4**	***f*+**	319 ± 4	
N	days	294		96		48		261		138		44	
